# A review on the origin of nanofibers/nanorods structures and applications

**DOI:** 10.1007/s10856-021-06541-7

**Published:** 2021-06-12

**Authors:** K. Chandra Babu Naidu, N. Suresh Kumar, Prasun Banerjee, B. Venkata Shiva Reddy

**Affiliations:** 1 Department of Physics, GITAM Deemed to be University, Bangalore, 562163 Karnataka India; 2 Department of Physics, JNTUA, Anantapuramu, 515002 Andhra Pradesh India; 3 Department of Physics, The National College, Bagepalli, 561207 Karnataka India

## Abstract

In this review work, we highlight the origin of morphological structures such as nanofibers/nanorods in case of various materials in nano as well as bulk form. In addition, a discussion on different cations of different ionic radii and other intrinsic factors is provided. The materials (ceramic titanates, ferrites, hexaferrites, oxides, organic/inorganic composites, etc.,) exhibiting the nanofibers/nanorods like morphological structures are tabulated. Furthermore, the significance of nanofibers/nanorods obtained from distinct materials is elucidated in multiple scientific and technological fields. At the end, the device applications of these morphological species are also described in the current technology.

**Figure:**
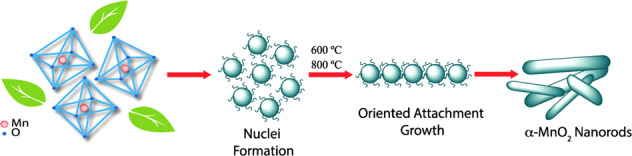
The nucleation and growth mechanism of α-MnO_2_ nanorods using natural extracts from Malus domestica and Vitis vinifera [3].

The nucleation and growth mechanism of α-MnO_2_ nanorods using natural extracts from Malus domestica and Vitis vinifera [3].

## Introduction

It is a known fact that nanotechnology is an innovative research area, and it develops various materials and devices on nanoscale (diameter of 10^−9^ m). To identify the nano ordered species within a material, the surface morphology of the same is a well-suited study. In addition, the morphology of different materials can be considered as an important parameter for achieving advanced properties. In view of this, ample of nanostructures such as nanospheres, nanoplates, nanorods, nanofibers, nanowires, nanoflowers, nanoleaves, nanotubes, nanocages, nanofilms, nanoparticles, nanosheets, nanochains, nanofoam, nanoholes, nanomesh, nanopillar, nanorings, nanoribbons, nanoshells, nanocubes, quantum wells, quantum dots etc., are evolved because of synthesizing nanomaterials via different synthesis techniques [1]. All these structures are very interesting and similarly, they provide peculiar properties along with scientific and technological applications. Moreover, the rapid development of science and technology is established owing to the formation of the above-said nanostructures.

Among all the nanostructures, the nanofibers/nanorods are different and the easiest structures to prepare. Many scientists put their efforts to develop nanofibers/nanorods like morphological species using electrospinning and hydrothermal techniques [2]. Out of these two methods, the electrospinning method is an expensive, time consuming, and laborious process [2]. On the other hand, the hydrothermal method offers several advantages like in expensive, less time consuming, easy preparation, and good homogeneity [2].

### Origin of nanofibers/nanorods like morphology

**Fig. 1:**
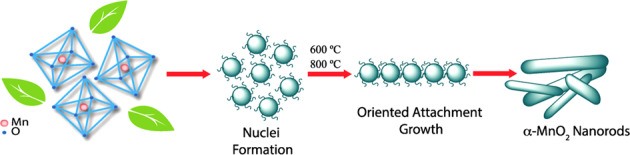
Schematic representation of the proposed nucleation and growth mechanism of α-MnO_2_ nanorods using natural extracts from Malus domestica and Vitis vinifera [3]

The nanofibers look to be flexible in nature while the nanorods will have rigidity in nature. Normally, the nanofibers/nanorods can be formed by the generation and growth of the nuclei of the material in vertical/horizontal direction. Besides, the heterogeneous nucleation will be reinforced at the solid interface owing to the deposition of catalytic clusters. During this kind of mechanism, the formed uniform nanopores can act as the templates for the nanofibers. The filled nanopores produce nanorods. This indicates that the nanorods will have apparent high dense structure rather than the nanofibers. The schematic representation for the formation of α-MnO_2_ nanorods via the nucleation process is shown in Fig. 1 [3]. After forming the nuclei, the nuclear clusters are being heated at 600 and 800^o^C temperatures. Consequently, some of the nuclei are oriented in direction while few are oriented in other directions. This leads to the formation of vertical/horizontal α-MnO_2_ nanorods (NRs). Similarly, the nanofibers will be formed containing the flexible (less rigidity) structure. It is a known fact that the nanorods show the different facets in the compound. This kind of behavior is indeed attributed due to the combination of ligands that act as the shape control agents offering different strengths to the nanorods. Therefore, various faces will be acquired by the nanorods with different growth and elongation rates. Herein, the ligand is a molecule/ion, and it can bind the metal atoms located at the central position in order to develop the complex system. It comprises Fe as the central metal ion position, K_4_ as the counter ion position, (CN)_6_ as the ligand, and [Fe(CN)_6_] as the coordination position. Moreover, these ligands are normally considered as the electron donors attracted O the Fe (metal atom) at the center of the complex system.

### Electrospinning technique

**Fig. 2:**
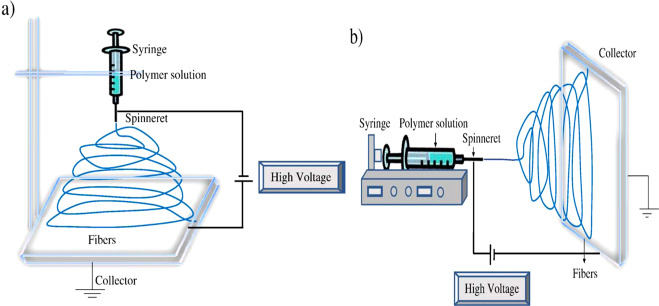
Schematic representation of nanofiber preparation with **a** vertical set up and **b** horizontal set up [4]

**Fig. 3:**
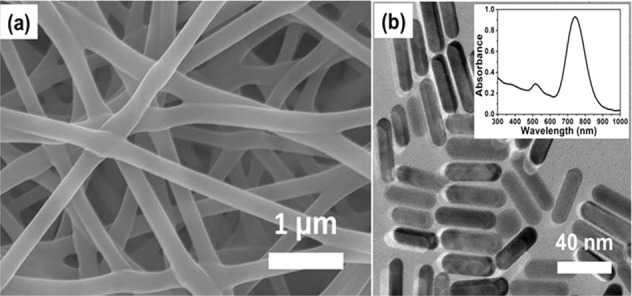
**a** SEM image of the PAA/PVA electro spun nanofibers; **b** TEM image and UV–vis spectrum of the as-prepared Au NRs [5]

Electrospinning is a synthesis technique of nanofibers with nano/micrometer size, wherein the electrostatic forces will be produced from the polymer-based material solution. For the preparation of nanofibers, the conventional spinning process is used as shown in Fig. 2 [4]. In this process, the DC input voltage is required to develop the electrospinning. Herein, the strong electrical repulsive forces dominate the weaker surface tension forces of the polymer material solution. Within the conventional electrospinning process, the vertical and horizontal techniques generate the nanofibers. These are conducted at normal atmospheric conditions. This system includes the high voltage power supply, spinneret, and collector. The high voltage is used to inject charge into the polymer material solution. In the meantime, the polymer solution is introduced into the capillary tube. In addition, it is to be taken care that the poisonous gasses will be released during the synthesis process. For this purpose, the ventilation chamber is provided to the whole system. At this moment, the electric field is switched on and then, the electrostatic repulsive forces will overcome the weak surface tension forces. As a result, the solution is accelerated towards the collector. On reaching the collector plate, the solvent will be evaporated leaving the polymer on the plate. Thus, the nanofibers will be formed on the plate which can be identified by the high resolved and sophisticated equipment such as FESEM and HRTEM. The PAA/PVA electro-spun nanofibers are shown in Fig. 3 [5]. It is understood that the nanofibers present in SEM picture show an apparent flexibility in nature. On the other hand, the TEM picture of as prepared Au NRs reveals the significant rigidity in nature owing to the less pore portion of the structure.

### Hydrothermal technique

**Fig. 4:**
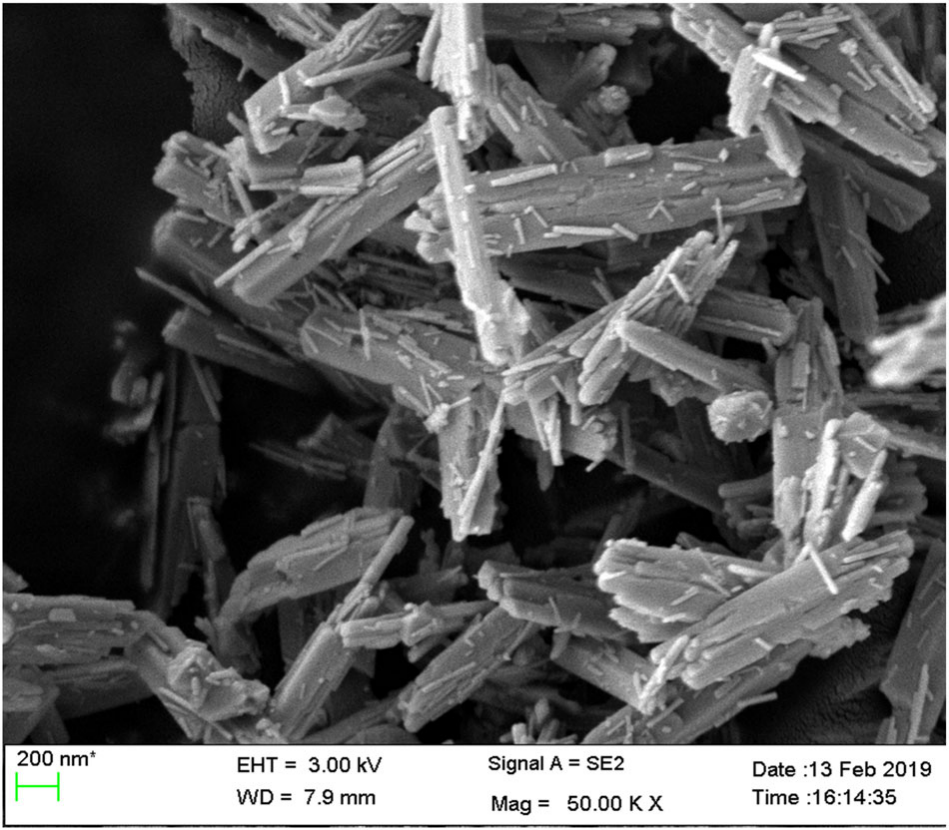
FESEM pictures of BaSrLaFe_12_O_19_ nanorods prepared via hydrothermal method [2]

**Fig. 5:**
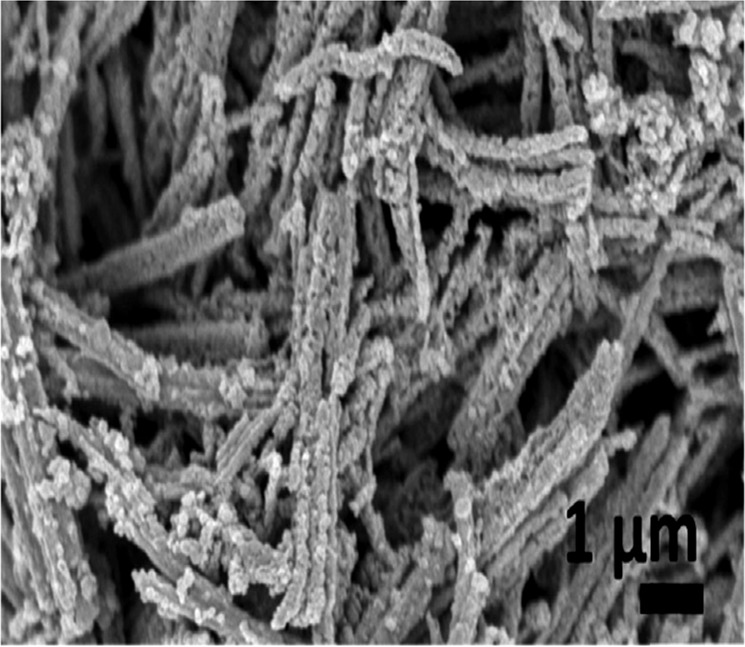
SEM picture of CoSe_2_/Mo_2_C/C nanofibers prepared via hydrothermal method [6]

The hydrothermal method is also preferable to prepare the nanofibers/nanorods. For this, several scientists considered the nitrate materials as the precursors. For instance, the BaSrLaFe_12_O_19_ nanorods are prepared via the hydrothermal process as. Herein, the nitrate precursors are selected as the starting materials. After dissolving the precursors in the distilled water, the NaOH solution is added drop by drop to maintain the pH value. The stirred solution is transferred to 300 ml autoclave and kept in an oven to perform the hydrothermal reaction at 150 °C for 8 h. In this method, the reaction temperature can be varied between 120 and 200 °C based on the kind of material compound. During the reaction, owing to the pressure and temperature developed in the autoclave, the nucleation process will be taken place. This will be occurred due to the presence of high and small ionic radii elements in the compound. In addition, different valence position of cations is also responsible for this kind of behavior. Once, the reaction is over, the sample is removed from the autoclave and then, it is washed multiple numbers times to keep the pH value neutral. Then, the powder sample is heated at 60 °C to remove the moisture content. The FESEM picture of BaSrLaFe_12_O_19_ reveals the pure nanorods (NRs) as shown in Fig. 4 [2]. Similarly, the SEM picture of CoSe_2_/Mo_2_C/C nanofibers prepared via the hydrothermal method is provided in Fig. 5 [6]. This picture evidence the flexible structure, wherein the pore portions are filled incompletely. The evolution of rectangular nanorods is originated from the substitution of La and Ba-elements of high ionic radii and different valence positions. Several reasons are observed for the formation of BaSrLaFe_12_O_19_ nanorods like the crystal structure, bonding preferences, kind of ions, pressure, temperature, surfactant, and pH as reported by Sunaina et al. [7]. In the literature also, Kumar et al. [8, 9], prepared the PLCT (Pb_0.8-y_La_y_Co_0.2_TiO_3_ (*y* = 0.2–0.8)), and PCLT (Pb_0.8_Co_0.2-z_La_z_TiO_3_ (*z* = 0.05–0.2)) samples via the microwave processed sol-gel method. The obtained results exhibited the nanofibers-like structures in the morphology. Similarly, the BCLF (Ba_0.2_Cu_0.8-x_La_x_Fe_2_O_4_ (*x* = 0.2–0.6)), and BCF (Ba_*x*_Cu_1-*x*_Fe_2_O_4_ (*x* = 0.2–0.8)) samples prepared via the hydrothermal method reveal the nanorod/nanofibers in the morphology as reported by Naresh et al. [10, 11]. BVS Reddy et al. [12, 13], synthesized the ALTBT nanocomposites consisting of the nanorods-like structures. Herein, the PLCT, PCLT, BCLF, and ALTBT nanostructures show the complete nanofiber/nanorods at high percentage of La^+3^ cations, whereas the BCF samples perform the same nature at high percentage of Ba^+2^ cations of high ionic radii. The similar observations are noticed by Prakash et al. [14], in case of BaCuLaTiO_3_ nanorods prepared using the hydrothermal method.

## Materials exhibiting the nanofibers/nanorods like structures and applications

It is a known fact that the materials are classified into several families based on the adapted structure. In view of this, the perovskites, spinels, nanocomposites, polymer composites, oxides, etc., are the popular materials achieving the nanofibers/nanorods like structures in the morphology. In Table1, the type of materials, synthesis method, applications etc., are represented. Lingmin et al. [15], prepared the ZnO nanorods/Ag nanofibers via soft solution and polyol process. The formed resultant structure was noticed to be hexagonal, wherein the optoelectronic gas sensor applications were obtained.

**Table.1:** Data on various nanorods/nanofibers materials and their parameters

Material	Synthesis method	Crystal structure	Diameter (nm)	Applications
ZnO nanorods/Ag nanofibers [15]	soft solution and polyol process	Hexagonal	-	optoelectronic gas sensor
Na_0.33_V_2_O_5_ nanorods [16]	one step annealing process	Monoclinic	400 nm	cathode for energy storage devices
Fe_2_O_3_/CNFs [17]	hydrothermal process	rhombohedral	20–40 nm	lithium-ion batteries
Pt/WO_3_ nanofibers [18]	two-step technique	Monoclinic	70 nm	photoelectrochemical
Bi_6_Fe_2_Ti_3_O_18_ nanofibers [19]	Electrospinning	orthorhombic	100–150 nm	photocatalysis
TiO_2_ nanofibers [20]	Electrospinning	Tetragonal	100 nm	FET devices
Cd-doped TiO_2_ nanofiber [21]	-	Tetragonal	200–330 nm	dye-sensitized solar cells
BiVO_4_/bi-phase TiO_2_ nanofibers [22]	electrospinning combined with hydrothermal	tetragonal/monoclinic	8–200 nm	photoelectrocatalytic
poly(3,4-proplenedioxythiophene) (PProDOT-(MeSH)_2_) nanofibers [23]	surfactant-assisted method	-	50–80 nm	electrocatalytic
Nitrogen doped graphite nanofibers (NGNF/MnO_2_) [24]	one-pot hydrothermal	Tetragonal	130–170 nm	Electrochemical detection of H2O2
porous hydrogen manganese oxide (HMO) nanofibers [25]	Electrospinning	Cubic	2–80 nm	lithium recovery
PAN/ZnO hybrid nanofibers [26]	Electrospinning	Hexagonal	800 nm	visible light photocatalysis
Au@PVP nanofiber [27]	sputtering	Hexagonal	410 nm	photodetector
ZnO-HT-PAN_H nanofibers [28]	-	Hexagonal	90-430 nm	antibacterial
polymer/biowaste derived carbon particles nanofibers [29]	-	-	91–123 nm	energy storage applications
ZnO nanofibers [30]	Electrospinning	Hexagonal	12–20 nm	solar cell
(1-x)(Al_0.2_La_0.8_TiO_3_)+(x) (BaTiO_3_)(x = 0.2-0.8) (ALTBT) nanorods [31]	Hydrothermal	cubic/tetragonal	8–17 nm	photocatalytic
TiO_2_ nanofibers [32]	Electrospinning	Tetragonal	8–100 nm	solar cell
CLCNF/PANi composite [33]	Electrospinning	-	-	supercapacitors
Si/TiO2/Ti2O3 composite carbon nanofiber [34]	Electrospinning	-	150 nm	Lithium-ion batteries
Nb_2_O_5_ NRs/NMMCNF film electrode [35]	Electrospinning	monoclinic	80–150 nm	electrochemical
Porous silica nanorods [36]	one-step hydrothermal acid-leaching	-	-	Functional material
Fe_2_O_3_ nanorod/carbon nanofiber [37]	electrochemical deposition	orthorhombic	-	lithium-ion batteries
PtNR-MCNF cathode [38]	Electrospinning	Cubic	300 nm	LiO_2_ batteries
graphene oxides (GOs) onto polyaniline (PANI) nanofiber [39]	in situ hybridization	-	63 nm	supercapacitors
RGO decorated Sb_2_S_3_ nanorods [40]	facile two-step process	orthorhombic	80 nm	sodium-ion batteries
CsPbI_3_ nanofibers [41]	Electrospinning	orthorhombic	300–600 nm	optoelectronic devices
α-Fe_2_O_3_/VGCF anode [42]	anodic electrodeposition	orthorhombic	16–21 nm	lithium-ion batteries
CNF//WO_3_ [43]	sodium chloride assisted hydrothermal process	Hexagonal	200–500 nm	supercapacitor
ZnO/SnO_2_ heterogeneous nanofibers [44]	Electrospinning	Hexagonal/tetragonal	70–120 nm	lithium-ion batteries
Mn_3_O_4_ nanofibers [45]	Electrospinning	Tetragonal	20–50 nm	supercapacitor
Li_2_MnSiO_4_ nanorods embedded in carbon nanofibers (LMS/CNFs) [46]	Electrospinning	orthorhombic	5 nm	lithium-ion batteries
AuNPs@NCNRs/CNFs [47]	in situ reaction	Cubic	160–280 nm	electrocatalyst for the hydrogen evolution reaction
PANi-P-1.0 [48]	plasma modification	-	180 nm	supercapacitor
NiCo_2_O_4_/CNF-450 [49]	Solvothermal	Rhombohedral/cubic	100 nm	pseudocapacitive performance
Graphene sheets (GNS) surface-grown with polyaniline nanorods (GP) [50]	interfacial polymerization	-	-	supercapacitor
HPCNF/MnO_2_ [51]	Electrospinning	monoclinic	300–400 nm	supercapacitor
Cu_2_O nanorods/nanotubes [52]	in-situ electrochemical technique	-	100–200 nm	non-enzymatic glucose sensing performance
PANI@CNFs composite [53]	one step of facile chemical oxidation	-	-	supercapacitor
SiO_2_@FeeN doped C nanofibers [54]	-	-	30–50 nm	lithium-ion batteries
carbon nanofibers (CNFs) @SnO_2_ nanocomposites [55]	co-electrospinning	Tetragonal	-	supercapacitor
PPy/WO_3_/BMIMPF6 [56]	electrochemical polymerization	-	-	Electrochromic applications
PVDF/KNN composite [57]	-	-	-	Piezoelectric
polymer nanofiber [58]	Polymerization	-	-	-
TiO_2_ nanorod [59]	Electrospinning	-	200 nm	solar cell
gold nanorods (NRs) [60]	-	-	20 nm	detection of molecules
Ni-Mo_2_C-CNF [61]	Hydrothermal	-	-	Renewable energy
ZnO nanorods [62]	Electrospinning	Hexagonal	-	catalytic
CdWO_4_ nanorods [63]	Hydrothermal	monoclinic	20-40 nm	blue-green luminescence
TiN nanorods [64]	Hydrothermal	-	130 nm	electrochemical
Ag/ZnO nanorods [65]	liquid precipitation citrate reduction process	Mixed	70-80 nm	Catalytic
Bi_2_S_3_/carbon nanofiber (CNFs) [66]	electrospinning and hydrothermal	orthorhombic	3.4 nm	liquid-state solar cells
BiCl_3_/PVDF nanofibers [67]	Electrospinning	-	-	piezoelectric
CNF@CuO NR [68]	low-temperature solution	monoclinic	15 nm	supercapacitor
Ag-doped TiO_2_ nanorods [69]	Electrospinning	-	35–40 nm	Photovoltaic
α-Fe_2_O_3_/PVA [70]	Electrospinning	orthorhombic	105–124 nm	electromagnetic pulse absorbers
Pt/Pd decorated p-type CuO nanorods [71]	Hydrothermal	monoclinic	50–60 nm	Gas sensors
Mo/N-doped TiO_2_ nanorods [72]	two facile steps	Tetragonal	-	organic pollutants degradation
NCF/Co_3_O_4_ [73]	Hydrothermal	Cubic	-	Lithium-ion battery
Co_9_S_8_/C-CNFs [74]	carbonization and sulphidation	Cubic	-	lithium-ion batteries
hydrogen-treated WO_3_ nanofibers [75]	Electrospinning	orthorhombic	-	cationic dyes removal from water
chitin nanofiber (ChNF) [76]	-	-	-	Material design
Cu_2_O nanorods deposited seaweed cellulose sheet [77]	Hydrothermal	monoclinic	-	pharmaceutical
gold coated poly (ɛ-caprolactonediol) based polyurethane/poly(N-isopropylacrylamide)-grafted-chitosan core-shellnanofibers [78]	Electrospinning	-	560 nm	cancer treatment
NFSiC and NRSiC [79]	Combustion	50-200 nm	-	biomedical
Fe_3_O_4_/polyacrylonitrile (PAN) nanofibers [80]	Electrospinning	-	200-400 nm	phenol removal in wastewater
poly (vinyl alcohol)/sodium hexametaphosphate nanofiber [81]	Electrospinning	-	-	recovery of Lanthanide ions from aqueous solutions
Pd-ZnO nanorod arrays [82]	wet-chemical	Hexagonal	50-200 nm	trimethylamine sensors
copperhexadecafluorophthalocyanine nanorods [83]	nanosecond-pulse laser fragmentation	-	20–40 nm	-
CuO nanorod [84]	Solvothermal	-	-	non-enzymatic detection of glucose
Au/ZnO nanofibers [85]	electrospinning and sputtering techniques	-	120–300 nm	photodetectors
Pr-modified ZnO nanofibers [86]	electrospinning-calcination	Hexagonal	180–330 nm	oxygen sensor
mesoporous In_2_O_3_ nanorod arrays [87]	one-step hydrothermal	Cubic	120–200 nm	ppb-level NO_2_ detection
α-Fe_2_O_3_ nanofibers [88]	Electrospinning	orthorhombic	200 nm	-
porous Co_3_O_4_/Cnanofibers [89]	dip-coating	-	2.4 nm	supercapacitors
Sb_2_S_3_@CNF [90]	Electrospinning	orthorhombic	10–50 nm	sodium-ion batteries
Ca-Ta_2_O_5_ nanorods [91]	Hydrothermal	Cubic	25–30 nm	biomedical
(PI/Ag)/ZnO-Ag [92]	electrospinning and hydrothermal	Hexagonal	200–600 nm	photocatalytic degradation
3D hierarchical carbon nanofibers/TiO_2_@MoS_2_ [93]	electrospinning, hydrothermal and in-situ growth	Hexagonal	500 nm	Energy storage
Polymers/ZnO nanorods [94]	Electrospinning	-	-	-
Co/SrCO_3_/CNF [95]	Electrospinning	orthorhombic	-	electrocatalyst
MnFe_2_O_4_ nanofibers [96]	Electrospinning	Cubic	54–374 nm	Energy storage
γ-Al_2_O_3_ nanorods [97]	solvothermal	orthorhombic	100–200 nm	-
alumina nanorods [98]	Hydrothermal	Cubic	200–300 nm	electrochemical
CsPbBr_3_/PS nanofibers [99]	Electrospinning	orthorhombic	-	LCD devices
NaBi(MoO_4_)_2_/Bi_2_MoO_6_/TiO_2_ nanofibers [100]	-	orthorhombic	10 nm	visible-photocatalysis
La(OH)_3_ nanorod [101]	Electrospinning	Hexagonal	50–310 nm	dephosphorization
NiCunanorods@carbon nanofibers [102]	Electrospinning	Cubic	-	dehydrogenation of ammonia borane
CoCr_7_C_3_ nanorods/CNFs [103]	Electrospinning	Hexagonal	-	electrocatalyst for methanol electro-oxidation
C@NiO/Ni nanofibers [104]	Electrospinning	Cubic	-	electrocatalyst for hydrogen evolution
Nickel nanorods/nickel foam [105]	Hydrothermal	Cubic	-	anode for direct alkaline methanol and ethanol fuel cell
Co_3_O_4_-loaded ZnO nanofibers [106]	Electrospinning	Hexagonal	100 nm	hydrogen sensing
Pd coated SnO_2_ nanofiber rods [107]	electrospinning and magnet sputtering	Tetragonal	-	hydrogen gas sensor
rGO-TiO_2_ composite nanofibers [108]	Electrospinning	Tetragonal	280 nm	solar cells
Fe_2_O_3_ nanorods/carbon nanofibers composite [109]	Hydrothermal	Rhombohedral	75 nm	lithium ion battery
MnO_2_ coated carbon nanofibers composites [110]	Hydrothermal	Tetragonal	400 nm	solar cells
Co/CNFs films [111]	Electrospinning	Cubic	500 nm	lithium ion battery
Au/TiO_2_ nanorods[112]	Hydrothermal	-	-	solar cells
Ta/TiO_2_ nanofibers [113]	Electrospinning	Tetragonal	40–60 nm	supercapacitor
Pt nanorods/polyamide-6 nanofibers templates [114]	Electrospinning	-	80–105 nm	electronic device
NiGa_2_O_4_ nanofibers [115]	Electrospinning	Cubic	50–150 nm	gas sensors
ɛ-iron oxide nanorods [116]	low-temperature aging	orthorhombic	400 nm	thermomagnetic
ZnO nanorods on ZnO nanofibers [117]	Electrospinning	Hexagonal	650 nm	photoresponse to UV and visible lights
TiO_2_slantednanorod arrays [118]	electron beam assisted physical evaporation	Tetragonal	-	Humidity sensor
SnO_2_ nanofiber/nanosheets [119]	Hydrothermal	Tetragonal	300 nm	formaldehyde detection
PAN/(PAN-b-PMMA) derived nanoporous carbon nanofibers loaded on ZnO nanostructures [120]	drop cast method	Hexagonal	87 nm	gas sensors
Pt-Cr_2_O_3_-WO_3_ composite nanofibers [121]	Electrospinning	monoclinic	500 nm	gas sensors

The varieties of nanorods or nanofibers have been prepared for different applications via different techniques. The ZnO nanorods and Ag nanofibers with hexagonal structures have been prepared on the chip via soft solution and polyol process. These nanomaterials are used as an optoelectronic gas sensor to identify the gases in surroundings such gas is commonly NO_2_, ethanol, CO, and CH_4_ with UV radiation assistance at the room temperature. The mere zinc oxide (ZnO) is not up to the mark to sense the gas at room temperature as the smaller number of electron carrier’s generation on the surface of the ZnO. Therefore, the silver (Ag) nanoparticles have been incorporated into ZnO structures to defeat the drawbacks so that to exhibit sensor application at the room temperature [15]. The one-dimensional vanadium oxide-based nanomaterials also exhibit gas sensor applications along with spin injection applications or model catalyst. The major advantages of oxides are many synthesis techniques which are sol–gel, hydrothermal, electrospinning, controllable self-assembly process. But the most effective method to prepare vanadium oxide is via annealing process. The high-temperature maintenance during annealing process morphological changes takes place and transition takes place from nanofibers to nanorods. Thus, formed nanorods have tens of microns in length and width around 400 nm [16]. The recent and major research is emphasized on the lithium-ion batteries in which the electrode materials play an important role in the storage of energy. The electrode materials are Fe_2_O_3_/CNFs which are synthesized by hydrothermal process and gives raise to rhombohedral structures with 20–40 nm of diameter. The iron oxides used in these batteries are nontoxic and eco-friendly in nature. In many research papers reported that the gravimetric and volumetric capacities 1000 mAh/g and 5300 mA h ml^−1^, respectively which are higher than the graphite electrodes. Lithium-ion batteries are the rechargeable and highest energy density batteries used in hybrid; plug in hybrid and electronic transport vehicles [17]. The Pt/WO_3_ and BiVO_4_/bi-phase TiO_2_ nanofibers exhibit photoelectrochemical and photo-electrocatalytic applications. In the two-step technique Pt/WO_3_ nanofibers could be synthesized with a monolithic structure with 70 nm diameter. The tungsten oxide is an n-type semiconductor with very narrow bandgap about 2.6 to 2.8 range and considered as a good photocatalyst. From the tungsten oxide nanorods, nanofibers and nanoplates can be prepared but the nanofibers are the most preferable because of high length to diameter ratio. The BiVO_4_/bi-phase TiO_2_ nanofibers prepared by the electrospinning combined with hydrothermal process gives tetragonal/monoclinic with a range of 8–200 nm diameters. The TiO_2_ based materials are eco-friendly and applied to decompose organic contaminants, reduction of carbon dioxide, and split water molecule into hydrogen [18, 22]. The Bi_6_Fe_2_Ti_3_O_18_ and TiO_2_ nanofibers are prepared by the electrospinning method. The diameters of the Bi_6_Fe_2_Ti_3_O_18_ and TiO_2_ nanofibers are 100-150 nm and 100 nm, respectively. The load of Au nanoparticles into Bi_6_Fe_2_Ti_3_O_18_ increases the photocatalytic activity used in the ferroelectric devices like multi-state memory devices, field sensing and catalytic activities. From the TiO_2_ nanoparticles we can prepare nanorods, nanofibers, nanobelts and nanosheets. Among these nanostructures the nanofibers are being preferred because of high surface to volume ratio, excellent electron transfer, and charge transferability. The famous applications of the TiO_2_ nanofibers are in the top-gate field-effect transistor. If the cadmium nanoparticles are doped with the TiO_2_, then its morphological structure becomes tetragonal with the diameter size 200–330 nm. These nanocomposites are used in the dye-sensitized solar cells [19–21]. The pendent thiol group grafed poly (3,4-proplenedioxythiophene) hallow nanofibers with diameter range 50–80 nm have an effective application in the electrocatalytic field and were prepared via surfactant-assistant method. These materials strengthen the electrochemical sensing ability due to their unique electronic transmission channels, effective molecular recognition ability and unique electrocatalytic properties [23]. The hydrogen peroxide (H_2_O_2_) plays important role in the biological systems, the required or balanced quantity of hydrogen peroxide is to be maintained. Hence to identify the hydrogen peroxide in the biological system we need to undergo electrochemical methods. In this method electrochemical sensors have been employed or adopted to analyze H_2_O_2_. The α-MnO_2_ nanorods grown on the NGNFs (nitrogen-doped graphite nanofibers) prepared by one-pot hydrothermal process with 130–170 nm diameter of nanorods and tetragonal structure are efficient electrochemical detectors of H_2_O_2_ [24]. The porous hydrogen manganese oxide (HMO) nanofibers play a vital role in the lithium recovery process from the seawater. The lithium is estimated in the sweater 2.5 × 10^14^ kg which is very low percentage (~0.17 mg/L) compared to other elements such as Na, Ca, Mg, and K. The recent studies and research have been concentrated on inorganic good adsorbent like highly porous hydrogen manganese oxide (HMO) nanofibers prepared via combining electrospinning, calcination, and ion exchanges. The HMOs will have cubic structure with diameter of 2–80 nm which have high capacity to absorb the lithium ions [25] (Table1).

Environmental hazardous are being increased in the globe due to the manmade and natural calamities. The chemical effluents are one of the major challenges to modern society and governments in the present context. Hence, the wastewater management is essential to scale down the hazardous outbursts. Some of the methods have been followed such as chemical oxidation, adsorption, photocatalysis, and membrane separation. Among these methods all are not cost effective and eco-friendly but photocatalysis is the cost-effective and greener technology with lower consumption energy. In the photocatalysis, the photocatalysts are the semiconductors with dopants or defect-induced or hybrid nanomaterials which can give higher photocatalytic action due to synergistic and plasmonic effect. The ZnO is n type semiconductor with non-toxicity, higher thermal and chemical stability with unique optical and photoelectrochemical properties. The ZnO has other important properties such as s high quantum yield and inherent surface defects which can sensitize under visible light. The most preferable nanostructure of ZnO is the nanofibers in this context as it provides high aspect ratio and a defined flow pattern for enhanced photocatalytic property. These nanofibers possess 800 nm of diameter with hexagonal structure prepared by electrospinning [26]. In the wearable electronics, stretchable and transparent conductors are essential to meet varieties of global demands without altering the resistance value. These conductors are made up of nanofibers Au metalized PVP prepared by sputtering which can withstand all types of mechanical deformations such as folding, twisting, bending, and stretching. These are synthesized in the form of nanofibers used in the memory parts of devices, electronic skins, electronic eye camera, and wearable health monitoring systems. The stretchable conductors are the important materials in stretchable electronics as they served as electrodes and interconnects in electronics system, particularly in optoelectronic devices along with stretchable solar cells, light emitting diodes, touch screens, and photodetectors [27]. The zinc oxide (ZnO) nanoparticles play very important in the antibacterial and dye removal activates. The Ag, Cu, TiO_2_, and ZnO nanoparticles are being used in the antibacterial agents and among these ZnO is the most effective nanomaterial in this connection. The ZnO is also used in the dye removal process effectively because of excellent adsorption coefficient by virtue of its surface morphology and volume to surface ratio. Hence the zinc oxide nanoparticles attached to polyacrylonitrile nanofibers with hinokitiol as gluing agent for synergistic antibacterial activities and effective dye removal [28]. And the ZnO nanofibers are prepared by electrospinning used in the solar cells. The carbon particles nanofibers derived from the bio-waste or polymer are being used in the energy storage devices [29, 30]. The aluminum-based nanocomposites like Al_0.2_La_0.8_TiO_3_ are the new trends in the field of photocatalytic applications. These nanocomposites are prepared by the hydrothermal process and depending on the composition the structure can be changed which is observed based on the X-ray diffraction analysis. This compound (1−*x*) (Al_0.2_La_0.8_TiO_3_) + (*x*) (BaTiO_3_) (*x* = 0.2–0.8) exhibits very good electrical properties and several advantages over other composites such as X-ray density (~11.210 g/cm^3^), surface area (~62.29 m^2^/g) electrical conductivity (~4.21E − 05 S/cm at 1 MHz) and dielectric constant of 62,768 at 1 kHz [31]. The TiO_2_ nanofibers are prepared by electrospinning used in dye-sensitized solar cells as an anode material. The TiO_2_ based materials are cost-effective and eco-friendly in nature [32].

**Fig. 6:**
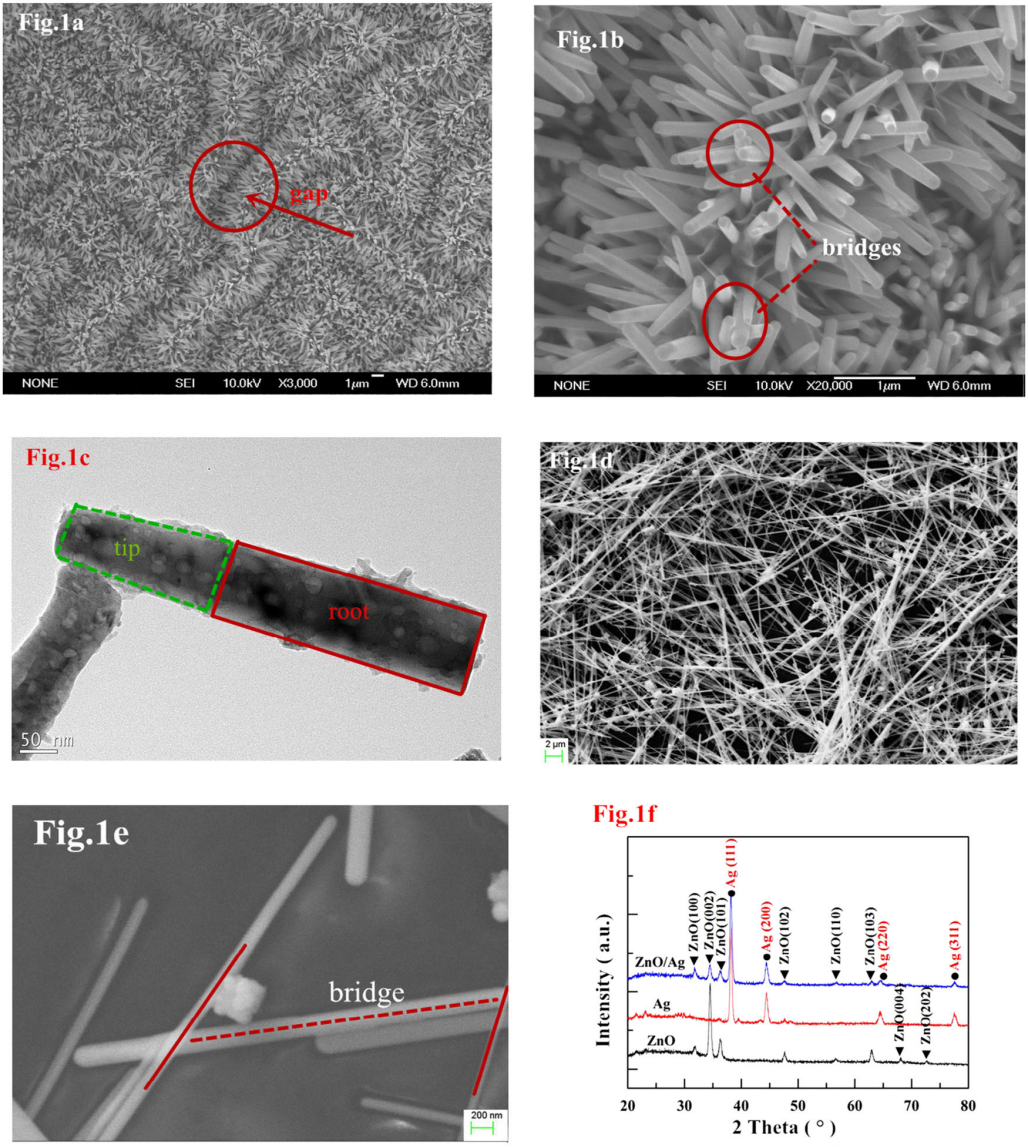
Low magnification (**a**) and high magnification (**b**) SEM of hierarchically nanostructured ZnO nanorods. **c** TEM image from one ZnO nanorod. The inset in **c** is a selected-area electron diffraction pattern. **d** SEM image of dense Ag nanofibers network. **e** Further magnified SEM image showing the bridging of Ag nanofibers. **f** XRD from ZnO nanorods, Ag nanofibers, and ZnO/Ag composites [15]

**Fig. 7:**
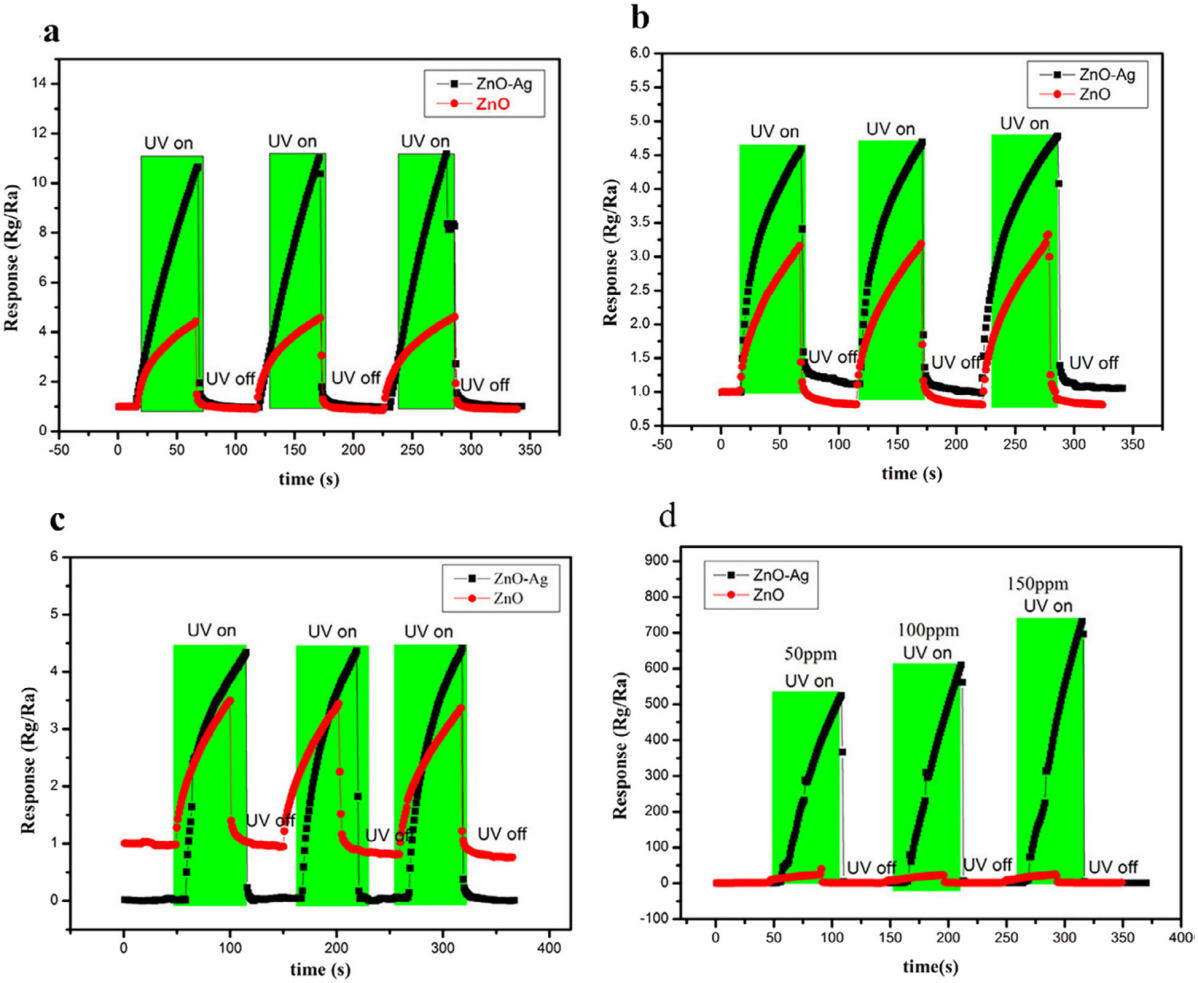
Response curves of the ZnO and ZnO/Ag composites upon exposure to 50 ppm of **a** CH_4_, **b** CO, and **c** ethanol, **d** different concentrations of NO_2_ under 365 nm UV Illumination [15]

It was a well-known fact that nanorods played a vital role in gas sensor applications. Lingmin et al. [15], prepared the pure ZnO nanorods and ZnO/Ag composite and showed the gas sensing performance. Especially, these devices exhibited the gas sensing activity for the C_2_H_5_OH, carbon monoxide, nitrogen dioxide, and methane after subjecting them to UV radiation. Based on the surface morphological behavior of these two samples, the gas sensing behavior was identified. That is, as indicated in the FESEM pictures of ZnO nanorods (Fig. 6 [15]), the multiple junction positions were noticed between the clusters of nanorods. These junctions can act as potential barriers. Therefore, there will be a clear formation of electrons and holes at these junctions. In addition, the doping of Ag into the ZnO system induced the formation of barriers. Due to the smaller value of work function of Ag than the ZnO, the electrons will be transferred easily from Ag portion to ZnO portion. Herein, the Ag–ZnO interface can act as the Schottky barrier trapping the electrons coming from the conduction band of ZnO. The resistance sensitivity response curves of both the samples were shown in Fig. 7 [15]. It was evident that the ZnO nanorods and ZnO nanorods/Ag nanofibers composites showed the gas sensing response under the UV-radiation illumination of 365 nm. Among these nanostructures, it was clear that the Ag nanofibers were acted as the barriers or bridge to the nanorods. Therefore, it was understood that the materials or composites possessing the nanorods/nanofibers like structures as well as the interface forces performed the efficient gas sensor applications.

**Fig. 8:**
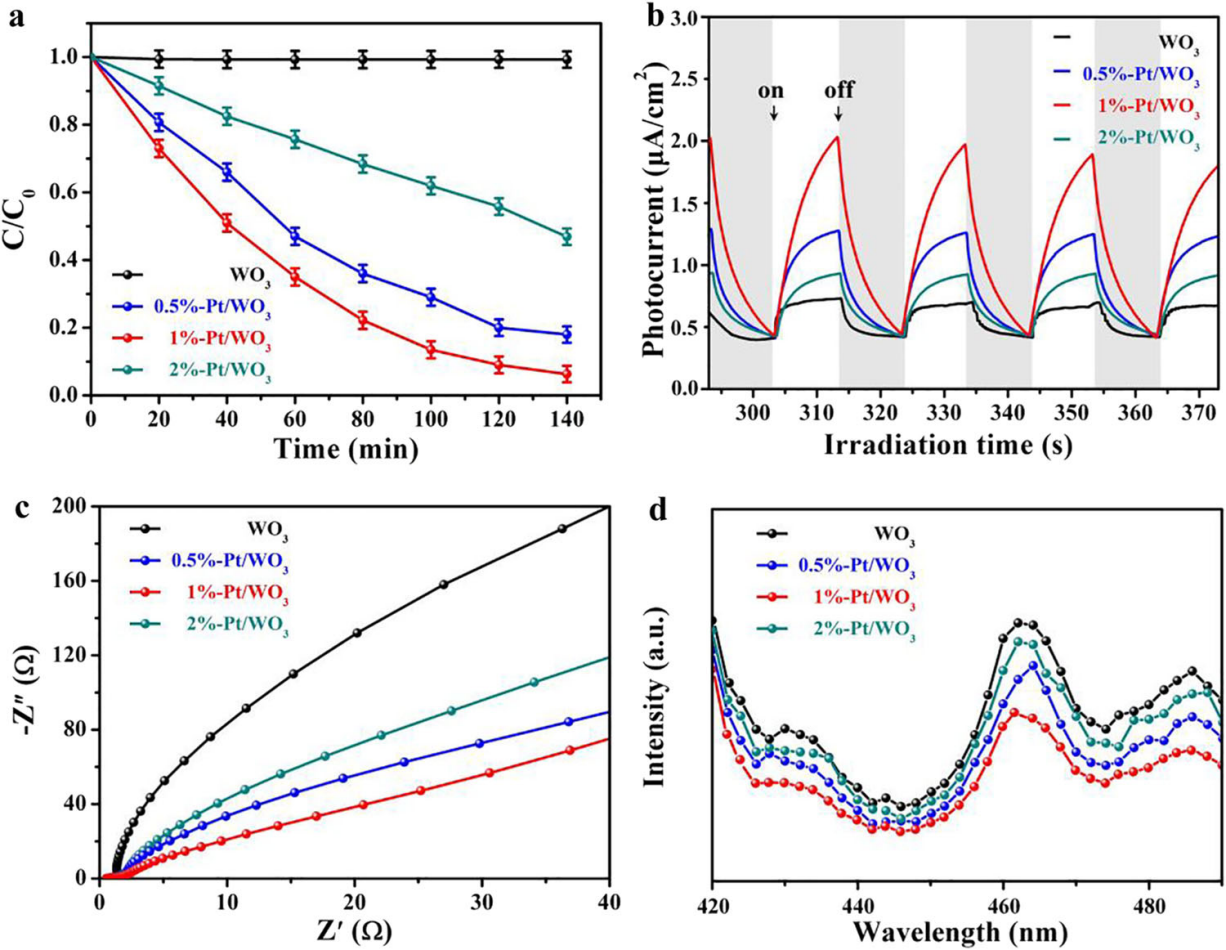
Photodegradation of RhB solution under visible-light irradiation (**a**), transient photocurrent responses (**b**), EIS Nyquist plots (**c**), and PL spectra (**d**) of Pt/WO_3_ nanofibers with different Pt contents [18]

**Fig. 9:**
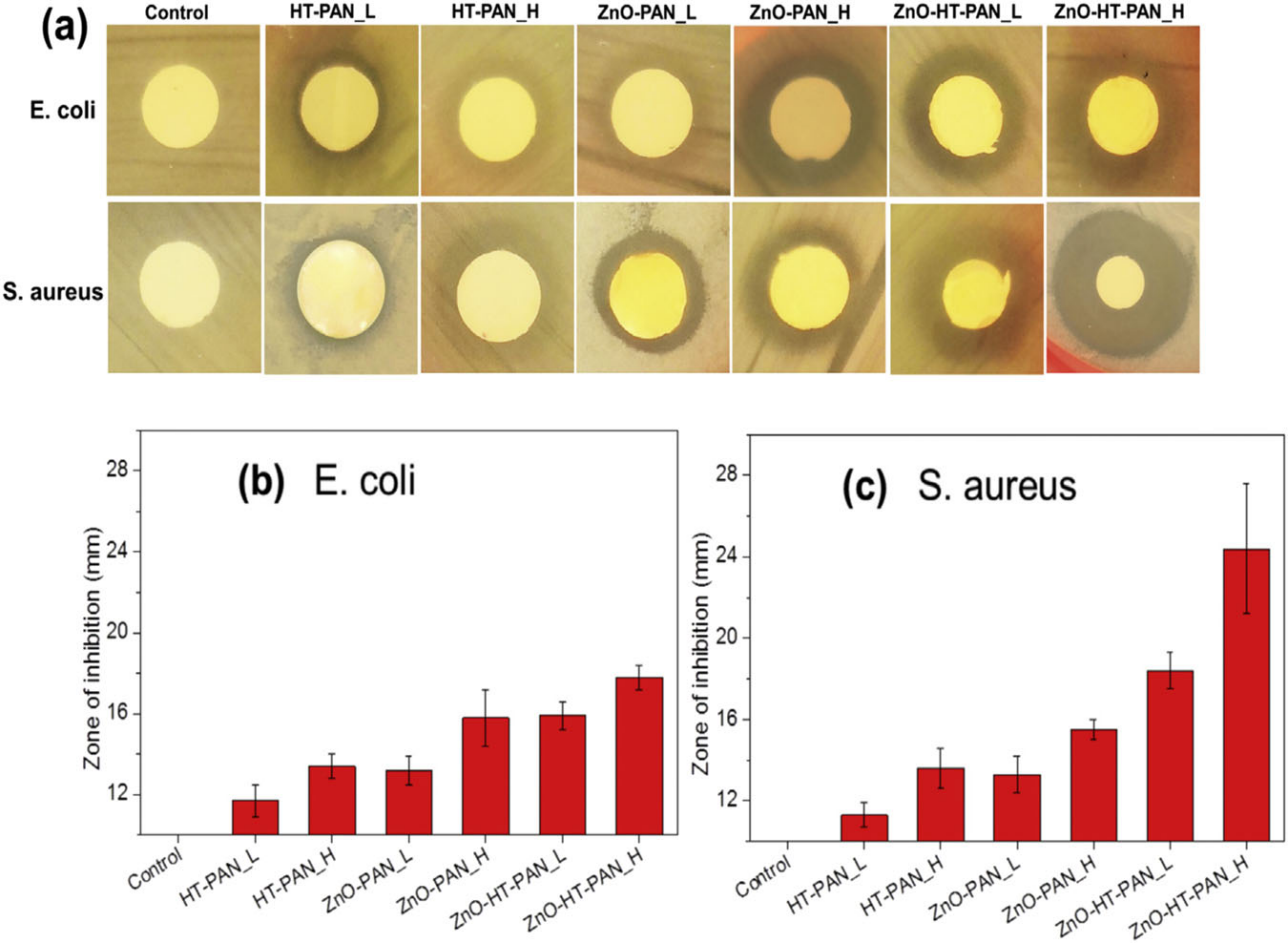
**a** Representative images of inhibition zones, **b** and **c** Calculated inhibition zones of nanofibrous samples based on disk diffusion test with standard deviations against E. coli and S. aureus [28]

In Fig. 8, it was observed that the Pt/WO_3_ nanofibers performed the photoelectrocatalytic activity as a function of various Pt contents. Herein, the bandgap was noticed to be decreasing with increase of Pt content. This evidenced a fact that the Pt nanoparticles can act as the Schottky barrier which can utilize the visible radiation and reinforce the generation and transfer of electrons and holes [18]. In addition, the photodegradation of RhB solution under the visible light irradiation clearly showed that the *C*/*C*_o_ ratio (Fig. 9a) was noticed to be decreasing with increase of Pt concentration and time. However, for the 1% of Pt, the photocatalytic activity was found to be high. This behavior was attributed to the uniform distribution of platinum nanoparticles on the surface of WO_3_ nanofibers and providing sufficient routes for electron transfer [18]. As a result, the 1% Pt/WO_3_ nanofibers offered the highest photocurrent density (Fig. 9b) as well as the smallest electrical resistance (high electrical conductivity) as compared with other samples. This was ascertained owing to the recombination of photogenerated carriers [18]. In Fig. 8c, the Nyquist plots of various concentrations of Pt/WO_3_ nanofibers were shown and it was understood that the relaxations were of non-Debye kind. Besides, the complete arcs were not formed due to the partial relaxation strength of carriers, wherein the carriers can move for the longer distances. The excitation wavelength of 464 nm was observed in the photoluminescence spectra of Pt/WO_3_ nanofibers (Fig. 8d). Especially, the low intensity was recorded for 1 % Pt/WO_3_ nanofibers, wherein the separation efficiency of photoinduced charges was high [18]. It was confirmed that with the incorporation of Pt nanoparticles into the WO_3_ nanofibers, some advanced properties were obtained as discussed above. In Fig. 9, the pure titanium dioxide nanofibers showed low current density while the 1 % Cd-doped titanium dioxide nanofibers offered the improvement of current density [21]. This indicated a fact that the moderate doping concentration of element induced the current density. This kind of manner was attributed to the photon-electricity conversion efficiency within the samples [21]. In reference [28], the antimicrobial activity was studied for ZnO/polyacrylonitrile nanofibers via the disk diffusion test method (Fig. 8a). It was clear that the inhibition zones were evidently noticed for ZnO/polyacrylonitrile nanofibers comprising of hinokitiol (HT) and zinc oxide (ZnO). Herein, the antibacterial activity of samples was performed against Escherichia coli (E. coli) and Staphylococcus aureus (S. aureus). It was noticed that the HT-PAN-L, HT-PAN-H, ZnO-PAN-L, ZnO-PAN-H, ZnO-HT-PAN-L, ZnO-HT-PAN-H (where PAN = polyacrylonitrile) offered a progressive inhibition zones against both bacterial strains (E. coli and S. aureus). These were shown in Fig. 9b, c. As a whole, it was confirmed that the combination of HT and ZnO induced stronger antibacterial activity against the S. aureus rather than the E. coli [28]. For all the samples, the inhibition zones were expanded significantly on combing the HT, ZnO, and PAN against E. coli and S. aureus. However, the similar combination showed the larger expansion of inhibition zones against S. aureus.

**Fig. 10:**
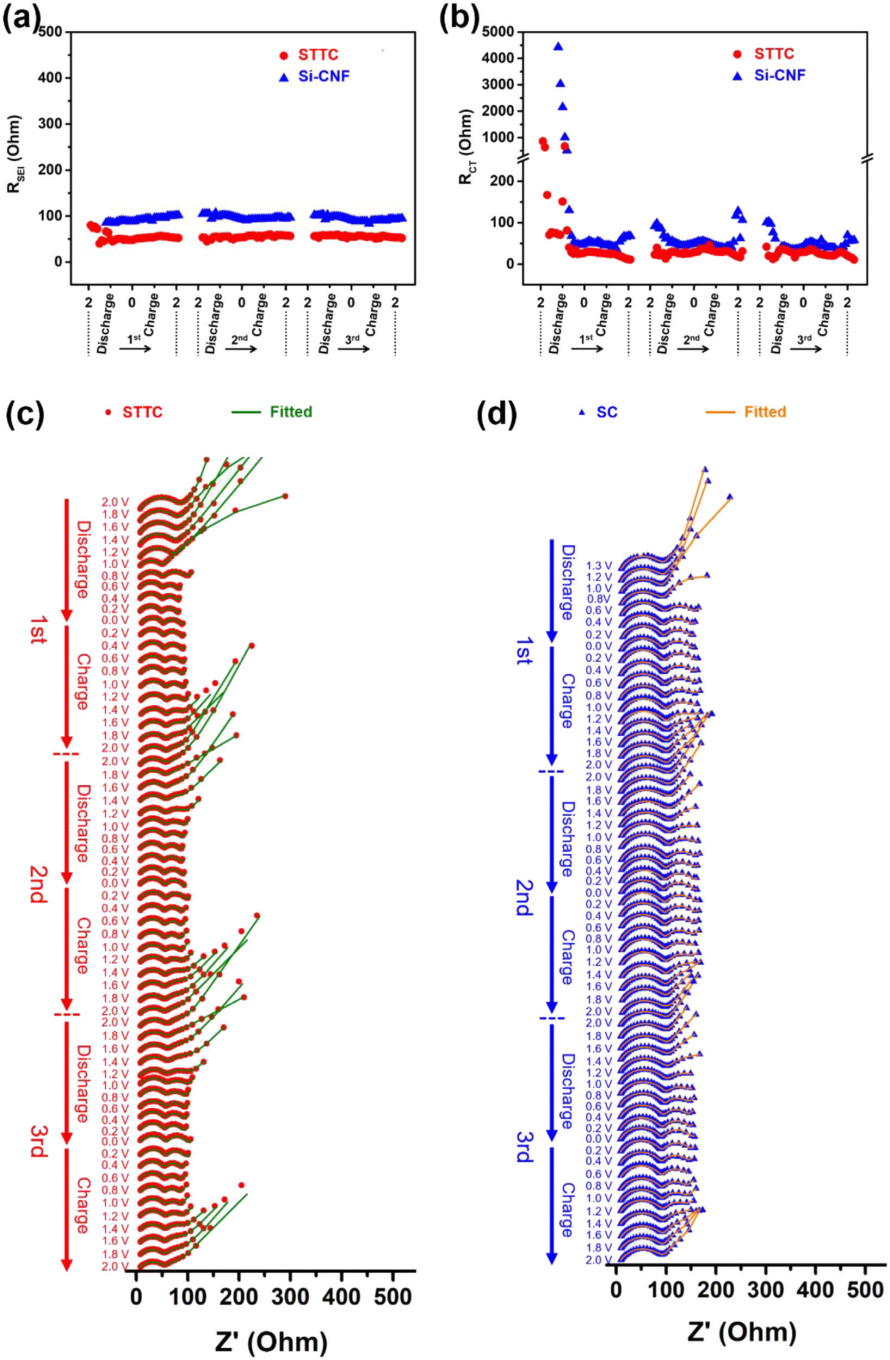
In-site SEI and Li-ion intercalation/de-intercalation in STTC and SC: fitted results of **a** solid electrolyte interface resistance (RSEI) and **b** charge-transfer resistance (RCT) simulated from the Nyquist plots. The *x*-coordinates in Fig. 6a and b represent voltage. Nyquist plots from in-situ EIS of **c** STTC and **d** SC electrode with the fitted curves calculated by the equivalent circuit shown in Fig. S14 [34]

**Fig. 11:**
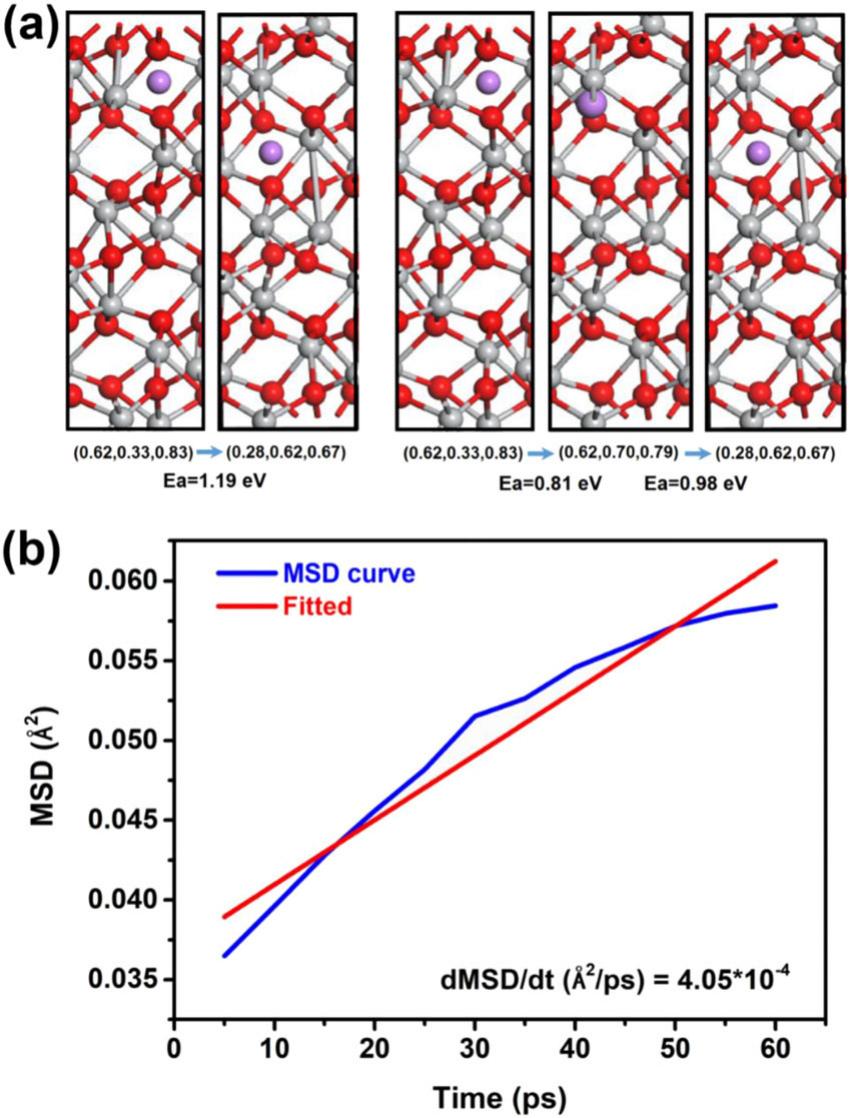
**a** Diffusion energy barrier based on density functional theory calculation, **b** Mean-squared displacements of Li ions in Ti_2_O_3_, and linear fit curve [34]

**Fig. 12:**
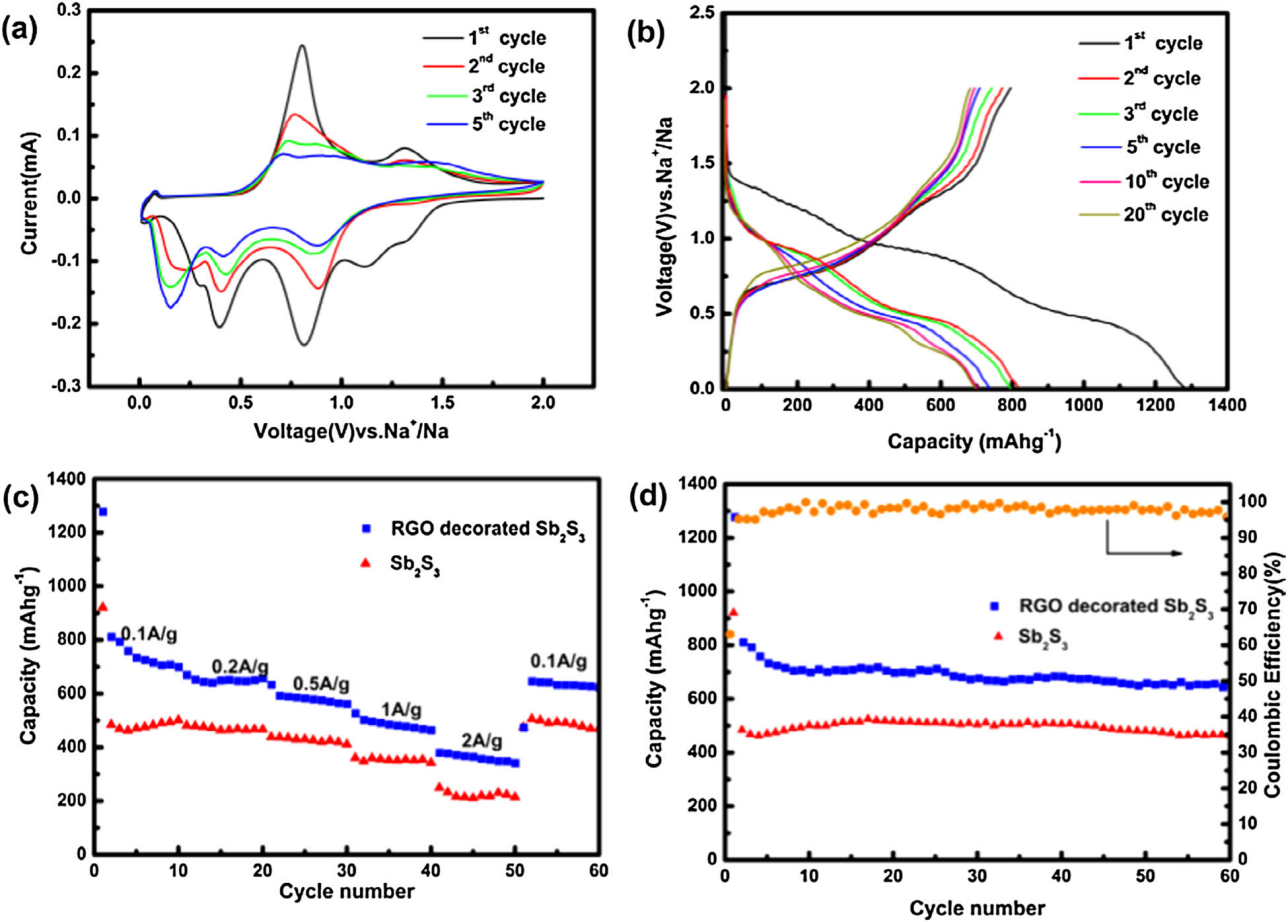
**a** CV curves of the first five cycles of RGO decorated Sb_2_S_3_ nanorods, **b** galvanostatic charge-discharge profiles of RGO decorated Sb_2_S_3_ nanorods, **c** rate capability of Sb_2_S_3_ and RGO decorated Sb_2_S_3_ nanorods, **d** cycling performances at 100 mA/g of Sb_2_S_3_, and RGO decorated Sb_2_S_3_ nanorods [40]

The electrochemical impedance spectroscopy (EIS) analysis of Si/TiO_2_/Ti_2_O_3_-carbon (STTC) nanofiber composites and SiC (SC) nanofibers were performed along with electrochemical kinetics as indicated in Fig. 10 [34]. This analysis was done using the in situ EIS analysis. Particularly, the Nyquist plots of STTC and SC nanofibers were shown in Fig. 10d, e. Two kinds of semicircular arcs were seen indicating the high and mid frequency arcs. The high frequency arcs were related to the solid electrode interphase (SEI) impedance (*R*_SEI_) while the mid frequency arcs were connected to the interphase charge transfer impedance (*R*_CT_). It was also clear that the *R*_SEI_ of STTC and SC nanofibers was almost constant at high frequency arcs for the first discharge cycle, whereas after the first discharge, the *R*_SEI_ (Fig. 8a) of STTC and SC (Fig. 8b) approached to 45 Ω and 90 Ω, respectively. The reason behind the constant trend of *R*_SEI_ can be attributed to the active particles which were perfectly coated by carbon nanofibers and therefore, it can reduce the evolution of SEI. Similarly, after first discharge, the surface of Si/TiO_2_/Ti_2_O_3_ and Si offered high-capacity retention and cyclic stability [34]. In addition, it was also noticed that for STCC, the *R*_SEI_ was almost constant while for SC, it was varied. The reason was the presence of titanium which works as buffer. But for Si, there was no buffer element. It was understood that the nanofiber structures and titanium buffer played a vital role in increasing the capacity retention and cyclic stability. Therefore, the STTC and SC were largely useful for the lithium-ion batteries applications [34]. To understand the diffusion of lithium ions in the Ti_2_O_3_, the diffusion coefficient (DC) was computed with the help of DFT and molecular dynamics model. The mean square displacement against the time (*t*) plots (Figs. 11, 12) indicated that the DC of lithium ions in titanium dioxide showed 10^−12^ cm^2^/s [34]. In addition, it evidenced that the Ti_2_O_3_ was developed in the surface portion of titanium dioxide. It became conductive to the transmission of lithium ions inside the silicon domains [34]. Fig. 11 indicates the diffusion energy barrier based on density functional theory calculation, and Mean-squared displacements of Li ions in Ti_2_O_3_, and linear fit curve.

In Fig. 12, the electrochemical behavior of reduced graphene oxide (RGO) coated Sb_2_S_3_nanorods was depicted [40]. Especially, Fig. 12a provides the cyclic voltammetry curves of RGO coated Sb_2_S_3_ nanorods for a step scan of 0.1 mV/s. It was found that the three reduction peaks were noticed at four voltages as indicated in Fig. 12a for the first cycle. This indicated the first discharge energy process. But on the other hand, the peaks were formed which were of relatively similar trend for all the next cycles. Herein, the formed two peaks were related to the reductive (at 0.85 V) and alloying (at 0.42 V) reactions. These anodic peaks were evolved owing to the reversible formation of Sb_2_S_3_ [40]. Similarly, the charge-discharge curves of RGO coated Sb_2_S_3_ nanorods (at specific density = 100 mA/g) showed two plateaus indicating the reductive and alloying reactions [40]. In addition, it was confirmed that the pure nanorods and RGO decorated nanorods showed the almost identical voltage performances. From Fig. 12c, it was clear that the pure Sb_2_S_3_ nanorods offered low-rate capabilities as compared to RGO decorated nanorods. The discharge capacity was decreased to 214 mAh/g at 2 × 10^3^ mA/g current density [40]. This evidenced a fact that the nanorod performance was increased little bit on coating it by RGO. The cyclic performance of RGO decorated nanorods showed high specific capacitances as compared with the pure nanorods at high cycles (Fig. 12d). For instance, the RGO decorated nanorods provided the discharge capacity up to 652 mAh/g after 60 cycles [40]. This alone revealed the improvement of discharge capacitance of RGO decorated nanorods rather than the pure nanorods. Therefore, the present materials were useful for the sodium ion batteries [40].

**Fig. 13:**
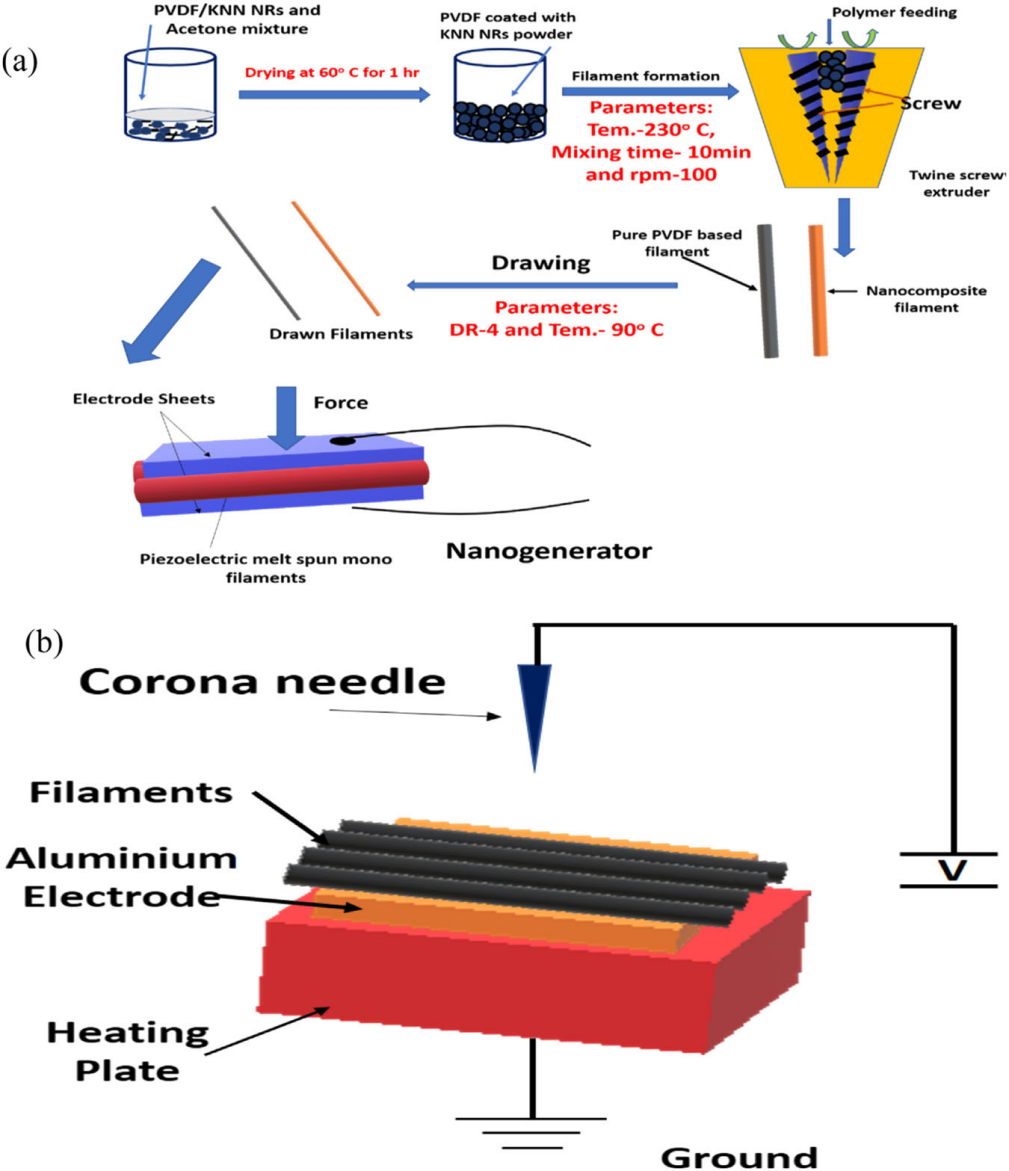
**a** Flow chart for melt-spun filament production and fabrication of nanogenerator and **b** Poling process of the melt-spun filaments [57]

**Fig. 14:**
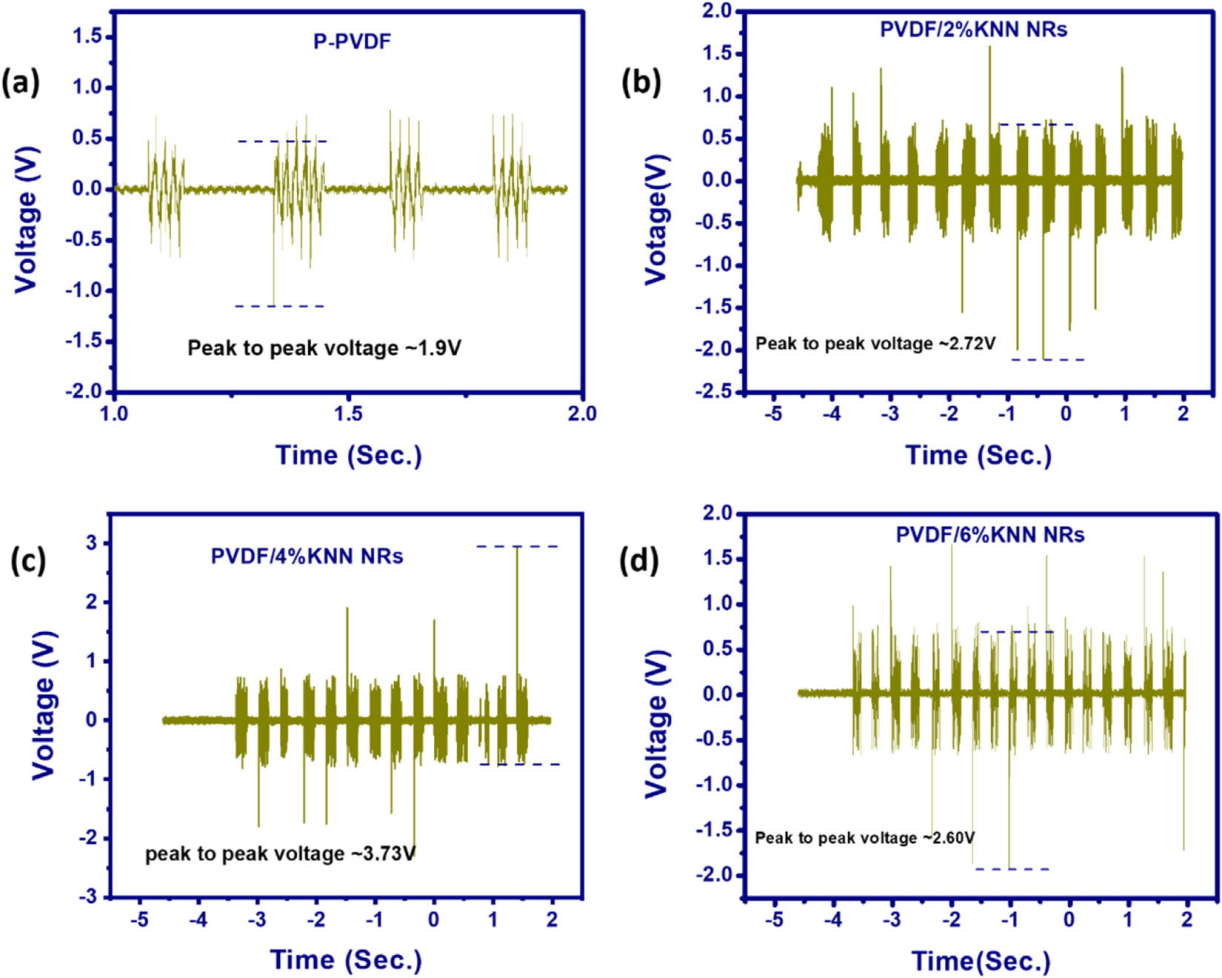
**a** Output voltage signal for the P-PVDF filament based nanogenerator, **b** Output voltage signal for the PVDF/2%KNN NRs filament based nanogenerator, **c** Output voltage signal for the PVDF/4%KNN NRs filament based nanogenerator, and **d** Output voltage signal for the PVDF/6%KNN NRs filament based nanogenerator [57]

**Fig. 15:**
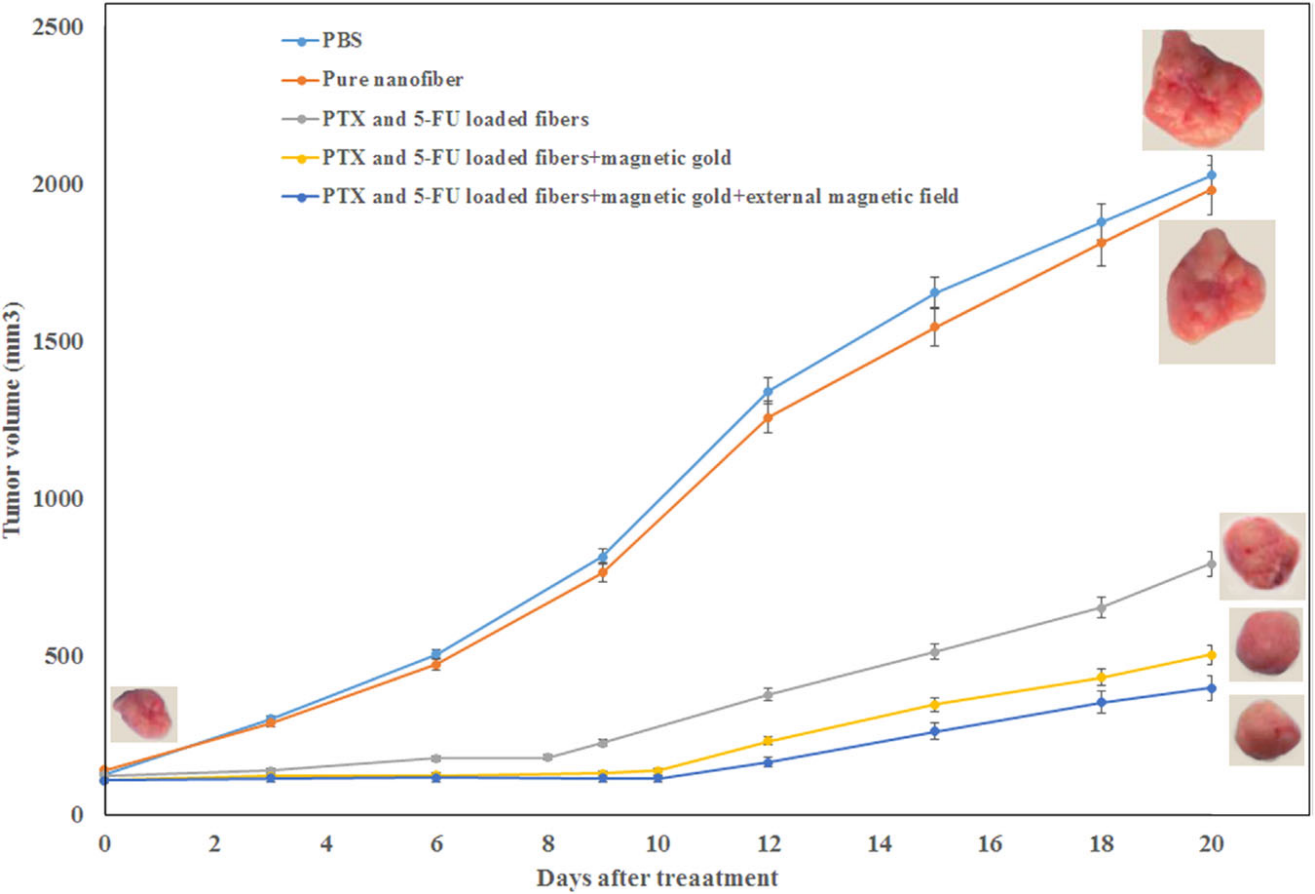
Tumor growth after treatment by the synthesized fibers [77]

**Fig. 16:**
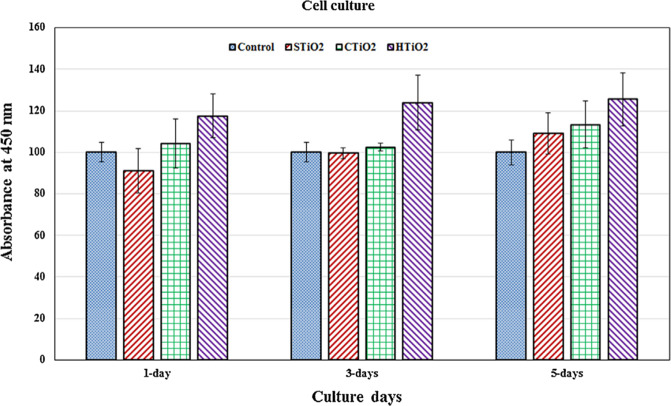
Cell culture results: absorbance of different materials through 1, 3, and 5 days of incubation [122]

In Fig. 13, it was seen that the composite nanofibers were prepared via different steps. That is, the PVDF pellet was coated with KNN nanorods powder of 0–6%, wherein the acetone was used as a dispersing agent [57]. The resultant pellets were kept in a twin-screw extruder. In Fig. 13a, the key parameters and processing conditions were clearly mentioned. As a result of these parameters and maintained conditions, the melt spun monofilaments were developed having an average thickness of 1 mm. Further, these filaments were kept nearer to the conductive aluminum tape for poling activity within the corona poling unit as shown in Fig. 13b. In addition, the poling was performed to the nanofilaments at 80 °C temperature for the DC voltage of 15 kV [57]. The piezoelectric behavior of nanogenerator prepared using different concentrations of KNN nanorods in PVDF pellets was provided in Fig. 14 [57]. Once the device was tapped using the finger, the output voltage can be found in the voltmeter. That is, if the force is applied on the surface of nanogenerator, the charge carriers will be away from their original positions and hence, they reach the top and bottom portions of the electrodes within the nanogenerator. Therefore, it develops a potential difference resulting the output voltage in the device. This voltage will be usually measured using the digital oscilloscope as mentioned in the reference [57]. The results ensured that the PVDF/4%KNN NRs filament based nanogenerator performed the highest output voltage of 3.7 V as compared with other concentrations. From the analysis, it was confirmed that the nanorods exhibited the piezoelectric behavior by producing certain output voltage. Thus, one can use these nanorods based materials for piezoelectric device applications [57]. In Figs. 15 and 16, the growth of tumor after treatment against the tumor volume was depicted. The antitumor efficiency of prepared nanofibers can be well understood using this experiment [77]. It was evident that the pure nanofibers have not showed much cytotoxicity on the cancer cells. At that time, even the tumor volume was increased to larger extent. Furthermore, the gold nanoparticles were added to the nanofibers and then the tumor growth volume was little bit reduced. This indicated a fact that the introduction of gold nanoparticles into the nanofibers allowed the cancer treatment as well. In addition, the magnetic field was applied to the system consisting of nanofibers plus gold nanoparticles. As a result of this, the growth of the cancel cell was come down to smaller extent. It was concluded that the additive species within the mixture reinforced reducing the tumor volume growth 18–20 days after treatment [77]. Fig. 16 shows the cell culture results: absorbance of different materials through 1, 3, and 5 days of incubation.

The nanofibers and nanorods are one dimensional nanomaterial which will have variety of applications in the medical or medicine field such as drug delivery, Cell patterning, cell differentiation, cell cluster formation, and biosensor. The structure itself is supplementary to the transportation in tubular structures of nanorods.

### Bone tissue regeneration

**Fig. 17:**
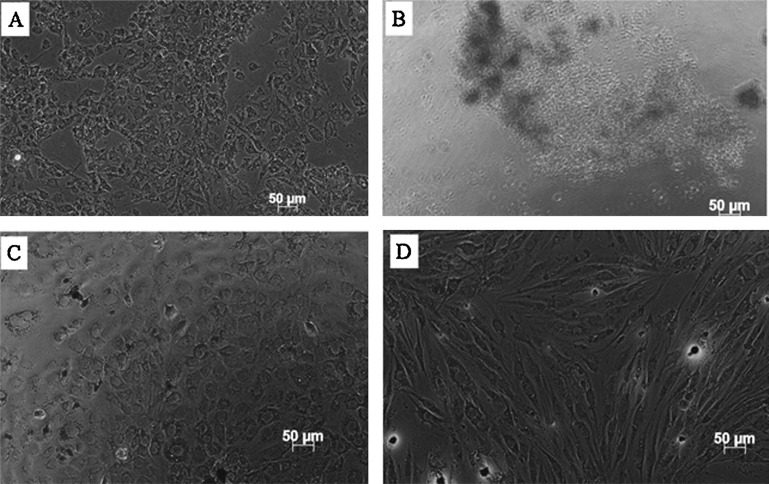
Microscopic cell morphology/proliferation after five days of incubation for **a** control, **b** STiO_2_, **c** CTiO_2_, and **d** HTiO_2_ [122]

**Fig. 18:**
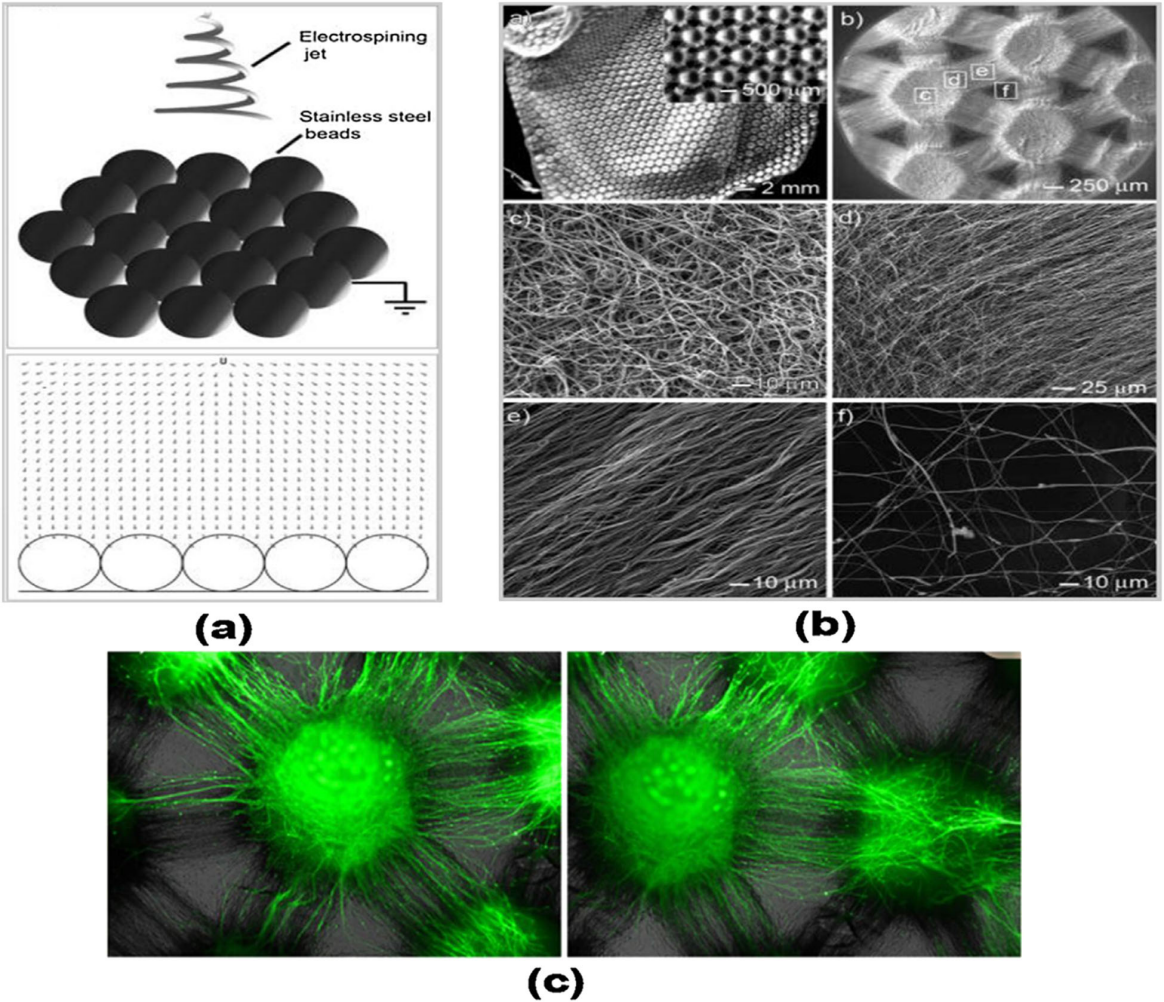
**a** Schematic representation of nanofiber membrane preparation, **b** optical micrographs and SEM images of nanofibers from different regions of microwell **c** fluorescence image of DRG neurites extended from one well to adjacent well after 6 days of culture [123]

High porous electro spun hollow titania (TiO_2_) nanofibers can be used in the bone tissue engineering [122]. TiO_2_ is one of the best nanomaterials in connection with biocompatibility due to its geometry, high aspect ratio and preserving the specific surface area on the level of reasonable porosity. In the bone, predominant inorganic material is the CaCO_3_ and calcium phosphate compounds. Due to emergence of 3D printing technology, which is coupled with computer-based CAD program, the artificial bones can be prepared. In the bone printing process, the mere calcium phosphate compounds do not give much mechanical strength as they are very low brittle in nature hence, we must add some nanoceramics into slurry to obtain high mechanical strength. The common ceramic materials are alumina, zirconia, and titanium. The titanium-based nanocomposite is superior for joint prostheses due to their appropriate compressive strength and biocompatibility than other ceramics. In terms of nano topographic effects on bone cell behaviors, TiO_2_ materials with various nanostructures display improved effects on development of bone tissue and bone regenerating capability. And the TiO_2_ nanotubes are expected to exhibit bone regeneration in medicine field and due to its porosity interaction between tissue and bone can be attained and achieve the transportation of the biomolecule between bone and tissue. Hence, the TiO_2_ supported hollow porous nanotubes functionalized with bioactive calcium salt might be a promising biomaterial for hard tissue replacement. The fabricated TiO_2_ immobilized cellulose acetate electro spun fibers prepared by electrospinning can be used in the photocatalytic degradation of textile effluents like methylene blue. The higher photocatalytic activity of fibers was discerned with high TiO_2_ content as CA composite ultrafine fibers with 5 wt% TiO_2_ were as high as 90% after 4 h of degradation [122, 123, 124]. It was also known that the proper combination of implant, and bone interface can become the vital aspect for implantation. In Fig. 17, the cell culture of various materials at various time intervals. The maximum absorbance of cell proliferation of hydrous titanium dioxide (HTiO_2_) was noticed from 1 to 5 days. In addition, the sulphonated titanium dioxide (STiO_2_) achieves the minimum absorbance for 5 days. However, the HTiO_2_ performed the good cell response at the end of incubation period. Further, it was also clear that the toxicity was completely reduced. Figure 18 was recorded to confirm the absorbance test. These micrographs show the cell morphology after incubation period. Herein, the minimum biocompatibility was observed in case of STiO_2_ nanofibers possessing the rounded cells. On looking at the performance of carbonated titanium dioxide (CTiO_2_) nanofibers, it was evidenced that these nanofibers revealed the significant cell spread rather than the STiO_2_ nanofibers. Moreover, comparatively, for HTiO_2_ nanofibers, the cell spread was improved with some dead cells on cell culture. The HTiO_2_ nanofibers showed good biocompatibility by providing the high cell spread due to the porous and hollow structure. Therefore, it can be well understood that the above strategies can improve the bone regeneration as well.

### Micropatterning technology in druyg delivery system

This technology is widely being used in the medicine field, where micropatterned nanofibrous can be used as scaffolds for biomedical application. This technology plays a vital role in the controlling surface microenvironment, cell-cell, and cell-tissue interaction. The micropatterning techniques are mainly based on surface chemistry (generation of patterns which consists of chemically different micro-domains) and micro-patterned areas (generated by physical barriers). Conventionally the micro patterns have been prepared by the photo or soft lithography. The micro-pattern and Electro spun nanofibers extensively have been used in the drug delivery system. Micro-structured and electro spun materials have large surface area which reduces the toxicity, enhanced drug encapsulation and increases the efficiency of delivery to the target. However, the behavior of drug delivery depends on the nature of the structure and reported that sustainable drug release can be achieved in nanofiber alignment and patterned fibrous scaffold. The release rate of fenbufen is slower in aligned Polylactic-co-glycolic acid/chitosan nanofibrous scaffolds compared to random fibers. The decrease of pore size in the aligned nanofibers reduces the delivery or diffusion rate than that of random fibers. Micro-structured cellulose acetate nanofibers by nylon mesh with 50–100 µm dimension openings showed a zero-order sustained drug release up to 12 h compared to nonpatterned scaffolds. The relevant reason is that minimization of solid-liquid interface and higher angle of contact controls the drug release. The hydrophobicity (depends on the nanofiber density and distribution pattern) of the substrate also alters the drug release. Polyvinyl butyral mesh patterns showed slower drug release due to the even distribution of nanofibers and higher contact angle. Multicompartmental fiber matrix and hydrogel microparticle were evaluated for the sustainable growth factor release [123].

### The cell differentiation

**Fig. 19:**
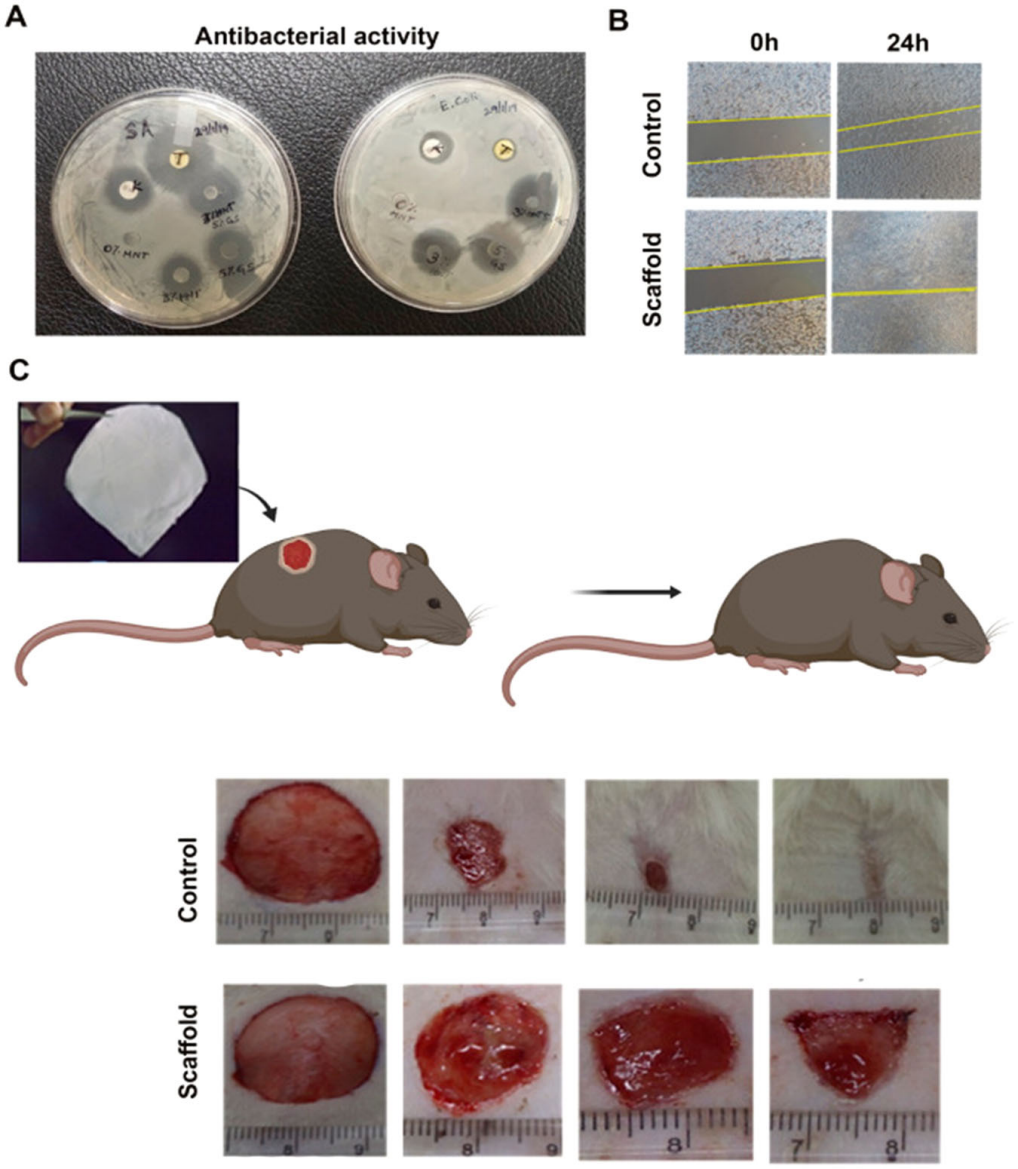
Antibacterial activity (A), in-vitro cell migration (B), and in-vivo wound healing experiments of the control and the scaffold [124]

The cell differentiation is one of the biological processes which depend on the many factors such as diffusible molecules, cell–cell interactions, cell–matrix interaction etc. The cell fate is affected by many factors including surface topography, surface chemistry, etc. The nanofiber orientation and micropatterned structure plays important role in the tissue engineering. The micropatterned polyethylene glycol hydrogel with gelatin fiber was considered for osteogenic differentiation. Fiber attached by MSC will be differentiated into osteogenesis due to two reasons such as sustained release of basic fibroblast growth factor and bone morphogenetic protein 2 up to 4 days and 4 weeks from the fiber and PEG hydrogel. The staining plays the crucial role in the osteogenesis differentiation due to the presence of alkaline phosphatase and alizarin red. Su-8 micropatterned nanotube of 20 µm dimension and grooved can induce self-early osteo differentiation without any external agencies. The cells rotate and align along the patterns with the respect to time. The three-dimensional nanofibers surface fabricated by cylindrical collector is twice greater than that of two-dimensional surfaces. Due to higher pore size in the three-dimensional nanofiber the cell infiltration and migration are higher. In addition, this scaffold showed osteogenic differentiation, which is correlated with the cell adherence, flattening, and spread. The patterned, Polycaprolactone nanofiber mesh which is from conducting wire net collector is reported as one of the best osteogenic differentiations in human bone marrow stromal cell [123, 124]. In Fig. 18, the schematic representation of nanofiber membrane preparation, micrographs, and the extended behavior of fluorescence image of DRG neurites after 6 days were provided (Fig. 19).

### Electro spun nanomaterials

These fibers will have different applications which are obtained from bio-macromolecules from botanical sources, polysaccharides from seaweeds, and polysaccharides from animal sources. The cellulose, starch and pectin are the bio-macromolecules and from these electrospinning patterns can be prepared by Electrospinning method. The cellulose is one of the good renewable biomaterial ecofriendly good mechanical strength and biocompatibility. And it has excellent ecological credentials with potential medical, pharmaceutical, paper production, sanitary products, rayon, cellophane derivatives, textile fibers, medical fabrics, and industrial applications. The major cellulose sources are the cotton, wood pulp and other pulps. The cellulose extracted from both plants and bacteria in the form of nanomaterials will have uniqueness over the microstructures vowing to excellent physical, mechanical strength, chemical reactivity and coupled with advanced technology like medical, catalytic, environmental, biosensory, and energy. In most of the cases the synthetic approach employed is top–down approach in the materials synthesis. The cellulose after undergoing electrospinning and obtaining the spun pattern of derivatives exhibits immobilization of biological active molecules or drug and vitamins such as alkannin, amoxicillin, curcumin, ibuprofen, indomethacin, naproxen, shikonin, sulindac, vitamin A, and vitamin E and these can improve the cell adhesion, proliferation and cell differentiation in the biological applications that too in the drug delivery and tissue engineering. In the tissue engineering, embedded gentamicin sulfate (impart antibacterial activity) and halloysite clay (increases mechanical properties) into cellulose ether-PVA nanofiber mats to deliver therapeutics to the skin tissue and scaffold composite heals the wound of in-vitro and in vivo. Similarly, polyaniline nanorods are preferred to transfer electro spun carboxymethyl cellulose and these are cylindrical and 3D surface materials which provides excellent electroactive surface which envisages electron transfer and mass transportation. Amino based cellulose synthesized by 6-deoxy-6-trisaminoethyl-amino (TEAE) cellulose and polyvinyl alcohol (PVA) blended solutions in aqueous media, and this can be used to stop the growth of S. aureus and this nanomaterial used to cure pneumonia which is caused by Gram-negative K. pneumoniae bacteria. Sodium doped cellulose with tungsten precursor on calcinations exhibits excellent gas (hydrogen disulfide) sensor applications. Starch is obtained from the higher plants used as a storage material and used in the food and nonfood applications. Starch consisting of complex polysaccharides α-D-glucopyranosyl units such as amylose and amylopectin found in wheat, maize, rice, potato, sago, tapioca/cassava, rye, barley, and oats. The starch with nanographene enhances electro spinnability, reduced diameter, improved hydrophilicity, and thermal stability. The starch derived nanotubes coupled with carbon nanotubes as an electrode are used in the rechargeable batteries which give rise outstanding performance. Pectin is obtained by the plants and it is hetero polysaccharide, and major sources are citrus peel, apple pomace, and sugar beet pulp. The pectin is used in the food industries as a thickening, gelling, and stabilizing agent and possesses biomedical applications [122, 123, 124]. Moreover, in Fig. 19, it was seen that the scaffold composite indicated faster in-vitro and in-vivo wound healing. The antimicrobial activity was increased on using the gentamicin.

## Summary and conclusions

The materials which are in nanoscale exhibit various structures like nanospheres, nanoplates, nanorods, nanofibers, nanowires, nanoflowers, nanotubes, nanocages, nanofilms, nanoparticles, nanosheets, nanochains, nanofoam, nanoholes, nanopillar, nanoribbons, quantum wells, quantum dots etc. Amongst, nanofibers/nanorods exhibit different characteristics. Owing to this, we have focused on recent advancements in nanofibers/nanorods. In this connection, this article provides the brief knowledge on the origin along with synthesis of nanofibers/nanorods by electrospinning and hydrothermal techniques. Further, various materials showed the nanofiber/nanorod like morphology along with their synthesis techniques, crystal structure and applications are tabulated. Finally, the device applications of nanofibers/nanorods such as nanogenerator melt spun filament and the role of nanofibers in the treatment of tumors are also discussed.
